# The prevalence of *Mycobacterium tuberculosis* using Gene Xpert among tuberculosis suspected patients in Gedeo Zone, Southern Ethiopia

**DOI:** 10.1186/s40001-022-00650-x

**Published:** 2022-02-12

**Authors:** Kuma Diriba, Gemechu Churiso

**Affiliations:** 1 Department of Medical Laboratory Sciences, Health Science and Medical College, Dilla University, Dilla, Ethiopia; 2 Department of Medical Laboratory Sciences, Immunology Unit, Health Science and Medical College, Dilla University, Dilla, Ethiopia

**Keywords:** Presumptive TB, MTB, Xpert-MTB/RIF assay, Ethiopia

## Abstract

**Background:**

Tuberculosis (TB) is a communicable disease remains a major global health problem and the leading cause of death from a single infectious agent. Even though many of the WHO recommended TB control strategies were implemented; there is still a major gap in TB case detection and treatment. This study aimed to determine the prevalence of *Mycobacterium tuberculosis* among presumptive TB patients in Gedeo Zone, Southern Ethiopia.

**Methods:**

A cross-sectional study was conducted on 384 TB suspected patients in Gedeo Zone from February to July 2021. Data were collected using a pretested structured questionnaire. Laboratory examination was processed using Xpert-MTB/RIF assay. Data entry was made using Epi info version 7 and analyzed by SPSS version 24. Logistic regression models were used to determine the risk factors.

**Results:**

Out of 384 study participants suspected with TB, *M. tuberculosis* was isolated from 103 giving an overall prevalence of 26.8%. Males (AOR) = 1.95; 95% CI 1.56–2.65, *P* = 0.01) were more likely to develop TB than females. Study participants who were illiterate (AOR 2.10; 95% CI 1.17–2.51, *P* = 0.014) were more likely to develop TB than the educated ones. Cigarette smokers (AOR 2.89; 95% CI 2.10–3.84, *P* = 0.01), khat chewers (AOR 2.86; 95% CI 1.28–3.79, *P* = 0.01), vaccination (AOR 0.52; 95% CI 0.21–0.88, *P* = 0.02), close contact (AOR 3.42; 95% CI 2.24–4.50, *P* = 0.01) and being positive for HIV (AOR 2.01; 95% CI 1.07–3.52, 0.01) were more likely to develop TB.

**Conclusion:**

Despite implementation of national and international TB control strategies, TB still remains one of the major public health problems in the country especially in the study area. The high prevalence of MTB was reported different risk groups. Early case detection and management of TB should be given special attention to strengthen and an appropriate control and prevention methods to reduce the emergence and increasing of MTB cases.

## Background

Tuberculosis is an airborne disease resulting from *M. tuberculosis*. TB can affect anyone anywhere. It typically affects the lungs and cause pulmonary TB [1]. The World Health Organization (WHO) recently announced that TB remains a major global health problem causing deaths among millions of people each year. TB is the ninth leading cause of death worldwide and the leading cause from a single infectious agent. Globally, TB kills almost three people every minute [2]. Globally, an estimated 10.0 million people fell ill with TB and 1.2 million died from the disease [2, 3]. TB mortality and incidence rate are falling at about 3% and 2% per year worldwide, respectively [4, 5].

According to the WHO report of 2019, more than 95% of TB deaths occurred in low and middle-income countries. Poverty may result in poor nutrition, which may be associated with alterations in immune function. It also results in overcrowded living conditions, poor ventilation, and poor hygiene habits which are likely to increase the risk of transmission of TB [6, 7]. WHO has been targeting an end TB strategy based on an assessment of the TB epidemic and progress in TB diagnosis, treatment, and prevention efforts. This shift in the approach to TB control, which includes among its 2030 targets (90% TB case detection and treatment) including in high-risk populations, and a cure rate of 90% of detected TB cases [8].

In Ethiopia various efforts have been made to control TB since TB was recognized as a major public health problem [4]. Despite all these national and international efforts, TB still remains one of the major public health problems in the country [5]. The recommended strategy to control TB in low income countries including Ethiopia, where 95% of the TB cases occur, is to detect and promptly treat smear-positive cases. It is known that delayed diagnosis results in more extensive disease, more complications and leads to a higher mortality. It also leads to an increased period of infectivity in the community [9].

Tuberculosis is exclusively transmitted based on environmental and personal associated risk factors [10, 11]. The risk factors contributing to acquiring TB infection are social and behavioral risk factors that include smoking, alcohol, khat chewing, and indoor air pollution [12]. Co-morbidities (people with certain chronic diseases) like diabetes, cancer, and HIV that affect the immune defenses system, close contact with active pulmonary TB patients, intravenous drug abuse, patients receiving immunosuppressive therapies and health care workers are those peoples at high risk of acquiring the TB infection [13–15].

The early diagnosis and treatment of TB patients is mandatory to reduce transmission of the disease. Millions of people are diagnosed and successfully treated for TB each year, averting millions of deaths, but there are still large gaps in detection and treatment. For the application of control policy, efforts for the identification of TB cases and treatment are mandatory which is not currently sufficient. As a result of this, updated knowledge of the prevalence of *M. tuberculosis* and their associated factor are crucial. Hence, the present study was intended to provide updated information on the prevalence of pulmonary tuberculosis and associated factor at Gedeo Zone, Southern Ethiopia.

## Methods

### Study design and study area

A cross-sectional study design was conducted from February to July 2021 in Gedeo Zone, Southern Ethiopia. The study populations were all pulmonary TB suspected patients who visited the selected health institution during the study period.

The study was conducted in Gedeo zone, which is located at southern direction of Ethiopia with a total estimated population of 1,694,868 according to the 2007 population census conducted by the Central Statistical Agency of Ethiopia (data are from Zonal health office). Gedeo Zone is found at a distance of 85 km from Hawasa and 365 km far from Addis Ababa, the capital city of Ethiopia. It is located in kola agro ecological zone with an altitude of 1400 km above sea level and annual temperature ranging from 22 to29 °C [16].

### Study population

The study populations were all pulmonary TB suspected patients of age ≥ 18 who visited the selected health institution during the study period. The inclusion criteria were all PTB suspected patients of age ≥ 18 years. The diagnosis of the patients was done by experienced physician and suspected cases for pulmonary TB with clinical manifestation of cough for two or more weeks, chest pain, or pain with breathing or coughing, night sweats, weight loss, fatigue, fever, chills, known or possible TB exposure were sent to laboratory for confirmation. The exclusion was patients with age < 18 years and study subjects who were unable to give informed consent were excluded.

### Sample size and sampling technique

The sample sizes was calculated using a single population proportion formula by taking the prevalence of *M. tuberculosis* (30.5%) conducted in Hawasa, 5% margin of error (*d* = 0.05) and 95% confidence interval (*z* = 1.96). The initial sample size was 349, and by considering a 10% nonresponse rate, the final sample size was determined to be 384.

### Data collection laboratory processing

Socio-demographic characteristics of study participants and clinical features were collected using pre-structured questionnaire and relevant data were collected from each study participant by trained health professionals. Those patients identified with signs and symptoms of pulmonary tuberculosis were ordered to bring a single sputum sample for the diagnosis of TB using Xpert-MTB/RIF assay (Cepheid, CA, USA). Briefly, 2 ml of Gene Xpert MTB/RIF sample reagent buffer was added to 1 ml of sputum specimen using a sterile pipette. The closed specimen container was manually agitated twice for 15 s and allowed to stand at room temperature for 10 min and again vortexed after 10 min and allowed to stand for 5 min and then 2 ml of the inactivated material was transferred to the test cartridge and the cartridge was then loaded into Gene Xpert device. Finally, the results were interpreted by the Gene Xpert diagnosis system from the measured fluorescent signals and displayed automatically after 2 h. Rapid HIV test was done according to the national HIV test algorithm [17].

### Data processing and analysis

After all demographic data and patients’ history were collected from the registration book; data entry, data analysis and data cleaning were done using Epi-Data 3.1 and SPSS version 23.0 software. Frequency count and percentage were used to present the findings. Prevalence figures were calculated for the total study population and separately by clinical features of the disease. Association factors were assessed by binary logistic regression and multivariate logistic regression. *P*-value less than 0.05 were considered statistically significant.

## Results

### Socio-demographic and clinical features of study participants

A total of 384 study participants suspected with TB aged ≥ 18 years were included in this study, out of which 64.3% were male and 35.7% were female with a sex distribution ratio of 1.8:1. The age of the study participants ranged from 18 to 91 years with the median age of 32 (interquartile range 25.7–51.3 years). Most of the study participants were found within the age range of 30–44 (41.6%). More than half (59.9%) of the study participants were from high school while those unemployed study participants account 54.9% (Table 1).

**Table 1:** Socio-demographic characteristics of study participant suspected with TB, Gedeo Zone, 2021 (*N* = 384).

Variables	Categories	Frequency	Percentage (%)
Gender	Male	247	64.3
	Female	137	35.7
Age categories	18–29	81	21.1
	30–44	160	41.6
	45–59	88	22.9
	> 60	55	14.4
Income	< 1500	122	31.8
	1500–3000	159	41.4
	> 3000	103	26.8
Residence	Rural	177	46.1
	Urban	207	53.9
Marital status	Single	206	53.6
	Married	165	42.9
	Divorced	13	3.5
Educational level	Illiterate	117	30.5
	High school or lower	230	59.9
	Collage and above	37	9.6
Occupation	Employed	173	45.1
	Unemployed	211	54.9

### Prevalence of MTB and different clinical features

Out of 384 study participant suspected for TB, *M. tuberculosis* was isolated from 103 giving an overall prevalence of 26.8%. 20.8% of study participants were smokers of 5 to 11 cigarettes per day while about one-third of study participants (31%) were khat chewers. In this study, again around one-third of TB suspected cases (32.3%) reported a history of close contact with known TB, while only 16.1% of cases reported as they were vaccinated for TB. In the current study, only 7.6% of study participants reported to be positive for HIV (Table 2).

**Table 2:** Personal life condition and comorbidity data of TB suspected study participants, Gedeo Zone, 2021 (*N* = 384)

Variables	Categories	Frequency	Percentage (%)
Smoke	Yes	80	20.8
	No	304	79.2
Khat chewing	Yes	119	31.0
	No	265	69.0
Vaccination for BCG	Yes	62	16.1
	No	322	83.9
MTB result	Detected	103	26.8
	Not detected	281	73.2
Close contact with known TB	Yes	124	32.3
	No	260	67.7
History of imprisonment	Yes	38	9.9
	No	346	90.1
Frequent alcohol	Yes	42	10.9
	No	342	89.1
Status of HIV antibody test	Yes	29	7.6
	No	355	92.4

### Analysis of contributing factors for *M. tuberculosis*

In bivariable analyses different age groups, residence, marital status, occupation, History of imprisonment and alcohol consumption had not shown an association with developing TB. In this study, males were more likely to develop TB than females (AOR 1.95; 95% CI 1.56–2.65). Study participants who had no educational background (AOR 2.10; 95% CI 1.17–2.51) were more likely to develop TB than the educated one. Cigarette smokers (AOR 2.89; 95% CI 2.10–3.84), khat chewers (AOR 2.86; 95% CI 1.28–3.79), vaccination (AOR 0.52; 95% CI 0.21–0.88), close contact (COR AOR 3.42; 95% CI 2.24–4.50), study participants who were positive for HIV (AOR 2.01; 95% CI 1.07–3.52) were more likely to develop TB (Table 3).

**Table 3:** Multivariate analysis of contributing factors among patients suspected with TB in Gedeo Zone, 2021 (*N* = 384)

Categories	MTB Pos *N* (%)	MTB Neg *N* (%)	COR (95% CI)	*P* value	AOR (95% CI)	*P* value
Gender						
Male	73(70.9)	174(61.9)	1.70(1.43–2.13)	0.045*	1.95(1.56–2.65)	0.01*
Female	30(29.1)	107(38.1)	1	1	1	1
Age categories						
18–29	22(21.4)	59(21.0)	1	1	1	1
30–44	42(40.7)	118(42.0)	1.16(0.55–2.46)	0.411	1.16(0.55–2.46)	0.401
45–59	24(23.3)	64(22.8)	1.0(0.51–1.99)	0.213	1.0(0.51–1.99)	0.103
> 60	15(14.6)	40(14.2)	1.05(0.51–2.18)	0.573	1.05(0.51–2.18)	0.363
Residence						
Rural	48(46.6)	129(45.9)	1	1	1	1
Urban	55(53.4)	152(54.1)	0.95(0.63–1.46)	0.521	0.95(0.63–1.46)	0.421
Marital status						
Single	55(53.4)	151(53.7)	1.43(0.44–4.63)	0.350	1.43(0.44–4.63)	0.350
Married	44(42.7)	121(43.1	0.73(0.23–2.34)	0.491	0.73(0.23–2.34)	0.491
Divorced	4(3.9)	9(3.2)	1	1	1	1
Educational level						
Illiterate	32(31.1)	85(30.2)	2.14(1.28–3.59)	0.016*	2.10(1.17–2.51)	0.014*
High school or lower	62(60.2	168(59.8)	1.88(0.83–4.24)	0.131	1.36(0.71–4.11)	0.130
Collage and above	9(8.7)	28(10.0)	1	1	1	1
Occupation						
Employed	46(44.7)	127(45.2)	1.09(0.72–1.68)	0.311	1.09(0.72–1.68)	0.243
Unemployed	57(55.3)	154(54.8)	1	1	1	1
Smoke						
Yes	21(20.4)	59(21.0)	2.23(1.12–3.57)	0.034*	2.89(2.10–3.84)	0.01*
No	82(79.6)	222(79.0)	1	1	1	1
Khat chewing						
Yes	32(31.1)	87(30.9)	2.66(1.28–3.89)	0.025*	2.86(1.28–3.79)	0.01*
No	71(68.9)	194(69.1)	1	1	1	1
Vaccination for BCG						
Yes	17(16.5)	45(16.0)	0.35(0.19–0.71)	0.023*	0.52(0.21–0.88)	0.02*
No	86(83.5	236(84.0)	1	1	1	1
Close contact with known TB						
Yes	33(32.0)	91(32.4)	2.63(2.24–4.46)	0.02*	3.42(2.24–4.50)	0.01*
No	70(68.0)	190(67.6)	1	1	1	1
History of imprisonment						
Yes	10(9.7)	28(9.9)	1.05(0.22–1.16)	0.108	1.05(0.22–1.16)	0.108
No	93(90.3	253(90.1)	1	1	1	1
Frequent alcohol						
Yes	11(10.8)	31(11.0)	1.24(0.65–2.38)	0.312	1.24(0.65–2.38)	0.211
No	92(89.2)	250(89.0)	1	1	1	1
Status of HIV antibody test						
Yes	8(7.8)	21(7.5)	2.91(1.06–3.51)	0.02*	2.01(1.07–3.52)	0.01*
No	95(92.2)	260(92.5)	1	1	1	1

## Discussion

Data on local epidemiology of MTB and associated risk factors were useful for the prevention and control of MTB, but limited data were available in the study area. In our study, the overall prevalence of MTB was 26.8%. In the current study, the prevalence of MTB was in line with previous reports from Hawasa 30.5%) [18], Debremarkos (23.1%) [19] and Nigeria, 22.9% [20]. However, our finding was lower than studies conducted in Congo, 79.1% [21] and Togo, 57% [22]. In contrast to our study, however, the study conducted in Amhara, 11% [23], Addis Ababa, 12.5% [24], South Africa, 13% [25] and Korea, 13.8% [26], Uganda 5.5% [27], and Oromia region, 3.8% [28] were lower than our current study. The difference might be due to variation in methodological techniques (culture vs. Xpert), study participants, study period, sample size, geography and TB control and prevention practices.

In this study, males were more likely to have MTB when compared to female patients. Similarly, studies conducted in Philippines [29], North Sudan [30] and elsewhere [19] reported as females were less likely to have MTB compared to males. The explanation for enhanced MTB in males could be probably due to males are mainly involved in outdoor activities and have more frequent contact with TB patients while females usually stay at home. In this study, there is no association between participants’ age groups, residence and marital status.

This is similar with studies conducted in different areas that reported no association between age, residence and marital status and TB infection [31–34]. However, different studies reported higher prevalence of MTB in age groups ranging from 16 to 34 years [35, 36].

In our study, TB patients who had no educational background (illiterate) were found to be more likely to develop TB compared to patients who had an educational background. Similar to our study, different studies reported that being illiterate was one of the contributing factors to develop TB [37–40]. Most of the communities living in developing countries were illiterate and have a low level of knowledge on TB. Low level of knowledge on TB can lead to complications and worse health outcomes, increasing the transmission and delaying health seeking behavior, lack of adherence, resulting in multidrug resistance, treatment failure, and disease complications and death [41, 42]. Thus, health programmers and stakeholder should give special attention and design a package in the national TB control program that addresses such areas where may people were lack awareness.

In the current study, smoking and khat chewing were found to be contributing factors for developing TB. This is in agreement with studies conducted in different areas which reported being smokers and khat chewers were risk factors for developing TB [38, 43–45]. Smoking damages the lungs and impacts the body’s immune system, making smokers more susceptible to TB infection. The occurrence of TB has been directly associated with impairment of the immune response and multiple defects in immune cells [46]. Smoking also results in histological changes in the lower respiratory tract, including peri-bronchial inflammation, fibrosis, vascular intimal thickening, and destruction of alveoli. This can be resulted in abnormal function of epithelial and damaged ciliary clearance of inhaled substances. Mechanical disruption of cilia function and hormonal effects could also appear secondarily to smoking [47–50]. Khat chewing is also associated with immune modulations that facilitates for TB development [51].

In our study, the occurrence of TB was less in those study participants who were vaccinated for BCG. This is in line with other studies conducted in different areas where lack of vaccination for BCG is a significant contributing factor for developing TB [39, 40, 52, 53]. The BCG vaccine is one of the most widely used of all current vaccines for neonates and infants in countries where it is part of the national childhood immunization program. BCG vaccination may be considered for health care workers who are employed in settings in which the likelihood of transmission and subsequent infection with *M. tuberculosis* strains resistant to isoniazid and rifampin is high. The protective efficacy of BCG for pulmonary TB in adults is uncertain [54, 55].

Our data demonstrate that close contact with known TB is one of the risk factors for the transmission and development of TB. Close contacts of patients with infectious tuberculosis are at increased risk of developing *M. tuberculosis* infection and disease [56, 57]. The prevalence of pulmonary TB in close contact was reported to be highest among many risk groups where there is an overcrowded population like homeless people, injection drug users, and prisoner live [58, 59]. Close contact is also one of the common risk factors for progression from latent TB infection to active disease [60]. Therefore, early diagnosis, isolation of known pulmonary TB and treatment is important to reduce and control the transmission of the disease.

In this study, HIV-positive patients were found to be contributing factors for the development of TB. HIV kills our immune system cells that help the body fight infections and diseases and facilitate for the development of TB. HIV and TB are considered as the double burden diseases of the world. According to WHO reports, there were 1.5 million deaths attributed to TB out of which 26% were due to HIV-associated TB [61, 62]. In under-developed countries, the prevalence of HIV is high and this resulted in an increased number of TB infections [63]. Of the 1.2 million TB–HIV cases worldwide, Africa accounts about 74% of the cases [63]. In Ethiopia, 4 in 100 people died due to TB–HIV co-infection and the incidence of multidrug-resistant tuberculosis (MDR-TB) was estimated to be 5.8 per 1000 people (64). In this study, being in prison and consumption of alcohol had no association with the development of TB.

### Limitations of the study

This study included a small sample size which may not be representative of a total population. Antimicrobial susceptibility tests were not assessed due to lack of infrastructure.

## Conclusion

The overall prevalence of MTB in this study was 26.8. Males were more likely to develop TB than females. Study participants who were illiterate were more likely to develop TB than the educated ones. Study participants who were cigarette smokers, khat chewers, BCG vaccination, close contact with known TB and being positive for HIV were more likely to develop TB. As the prevalence of MTB was still high despite the implementation of national and international TB control strategies and no significant reduction from time to time; health programmers and stakeholders should give special attention on early case detection and management of TB to strengthen and an appropriate control and prevention methods to reduce the emergence and increasing of MTB cases and design a package in the national TB monitoring and evaluation of the program that addresses such areas where thousands of people are live in overcrowded area.

## Data Availability

All data relevant to the study are included in the article and other raw dataset used for analysis during the current study are available from the corresponding author on reasonable request.

## Abbreviations

AFB: Acid-fast bacilli
AOR: Adjusted odds ratio
COR: Crude odds ratio
EPTB: Extra-pulmonary tuberculosis
HIV: Human immunodeficiency virus
MDR: Multi-drug resistance
MDR-TB: Multidrug resistant tuberculosis
MTB: *Mycobacterium tuberculosis*
PTB: Pulmonary tuberculosis
RIF: Rifampicin
RR-TB: Rifampicin-resistant tuberculosis
RR-MTB: Rifampicin-resistant *Mycobacterium tuberculosis*
SSA: Sub-Saharan Africa
TB: Tuberculosis
WHO: World Health Organization
